# The prevalence and risk factors of sleep disturbances among mental health patients following hospital discharge

**DOI:** 10.3389/fpubh.2025.1595303

**Published:** 2025-07-30

**Authors:** Wanying Mao, Reham Shalaby, Ernest Owusu, Hossam Eldin Elgendy, Belinda Agyapong, Ejemai Eboreime, Peter H. Silverstone, Pierre Chue, Xin-Min Li, Wesley Vuong, Arto Ohinmaa, Valerie Taylor, Andrew J. Greenshaw, Yanbo Zhang, Vincent I. O. Agyapong

**Affiliations:** ^1^Department of Psychiatry, University of Alberta, Edmonton, AB, Canada; ^2^Department of Psychiatry, Dalhousie University, Halifax, NS, Canada; ^3^Clinical Operations Informatics Office, Alberta Health Services, Edmonton, AB, Canada; ^4^School of Public Health, University of Alberta, Edmonton, AB, Canada; ^5^Department of Psychiatry, Cumming School of Medicine, University of Calgary, Calgary, AB, Canada

**Keywords:** sleep issues, psychiatry discharge, mental health, prevalence, risk factors

## Abstract

**Background:**

Sleep disturbances significantly impact psychological wellbeing, particularly during the critical transition when patients are discharged from psychiatric units. Despite extensive research on sleep and mental health, limited attention has been given to this transitional period.

**Aim:**

This study examined the prevalence and risk factors of sleep disturbances among patients preparing for discharge from psychiatric units in Alberta, Canada.

**Methods:**

This cross-sectional epidemiological study involved face-to-face interviews with eligible patients, followed by an online survey assessing sleep issues using the Patient Health Questionnaire (PHQ-9). Additional data on demographics, clinical information, and responses to the Generalized Anxiety Disorder (GAD-7) and World Health Organization Well-Being Index (WHO-5) were also collected.

**Results:**

Of the 1,437 patients approached, 1,106 participated. The prevalence of sleep disturbances was 79.6%. Key factors associated with sleep issues included relationship status (Chi2 = 13.39; *p* = 0.01), primary mental health diagnoses (Chi2 = 61.35; *p* < 0.001), anxiety (Chi2 = 80.28; *p* < 0.001), and poor wellbeing (Chi2 = 82.18; *p* < 0.001) at baseline.

**Conclusion:**

The study reveals a high prevalence of sleep disturbances among patients preparing for discharge and identifies key risk factors. These findings underscore the need for targeted interventions to address sleep-related issues during the discharge transition, improving recovery outcomes and reintegration into the community.

## 1 Introduction

Sleep is a cornerstone of wellbeing, and sleep disorder is a significant global public health concern, and adequate sleep is essential for the brain and body to recover from daily stressors, directly influencing mental and physical health (1). Sleep disorders are highly prevalent, with the *International Classification of Sleep Disorders* identifying approximately 90 distinct conditions, characterized by excessive daytime sleepiness, difficulty initiating or maintaining sleep, and abnormal movements or sensations during sleep (2). Poor-quality or insufficient sleep impairs daily functioning, adversely affecting work, relationships, and family life. Additionally, it is associated with increased risks of chronic conditions such as diabetes, obesity, cardiovascular diseases, cancer, mental health disorders, and mortality (3).

Recent findings underscore the widespread nature of sleep disturbances. For instance, nearly 30% of adults in the United States report difficulties with sleep initiation or maintenance, and over 27% experience excessive daytime sleepiness (4). Similarly, in Canada, more than one-third of individuals aged 5–79 fail to achieve the recommended daily sleep duration (5). Among Canadian adults aged 18–79, one-quarter report persistent difficulties with sleep, and 10% of children and adolescents experience frequent sleep-related issues (5). Numerous factors have been identified as potential predictors of sleep disturbances, including female gender, physical illnesses, African-American ethnicity, low socioeconomic status, engagement in manual labor, widowhood, marital quality, loneliness, perceived stress, preclinical dementia, prolonged use of benzodiazepines and sedatives, low testosterone levels, and elevated inflammatory markers (6) Among these, mental health disorders consistently emerge as the most significant and reliable risk factor (6).

Extensive research has demonstrated that brain activity during sleep is fundamental to maintaining mental and emotional wellbeing, and sufficient high-quality sleep facilitates the brain’s ability to process emotional information, evaluate experiences, and consolidate thoughts and memories (7). Critically, inadequate sleep appears to impair the consolidation of positive emotional content, underscoring its importance in emotional regulation (7, 8). Sleep disturbances are notably prevalent among individuals with psychiatric and mental health disorders (9). According to a report from Harvard Medical School, between 50 and 80% of psychiatric patients experience chronic sleep disturbances, compared to 10–18% of the general population (10). Sleep dysfunction is particularly pronounced in individuals diagnosed with anxiety, depression, bipolar disorder, and attention deficit hyperactivity disorder (ADHD) (10). This bidirectional relationship between sleep and mental health highlights how sleep disruptions can exacerbate psychiatric symptoms, while mental health conditions can further aggravate sleep disturbances, creating a perpetuating cycle with significant implications for overall wellbeing (7–9).

While the relationship between sleep disturbances and mental health is well-documented, particularly during active treatment phases, limited research has explored the critical transitional period when patients are discharged from psychiatric units (11). This study addresses this gap by examining the prevalence and predictors of sleep disturbances among patients during discharge from psychiatric care in Alberta, Canada. By focusing on this vulnerable phase, the study aims to identify key factors contributing to sleep disturbances and highlight opportunities for targeted interventions. This novel approach is essential for improving recovery outcomes, supporting patients during this transitional period, and enhancing their long-term quality of life.

## 2 Methodology

### 2.1 Study setting and design

This study was conducted in Alberta, Canada, a province with an estimated population of 4.7 million. Participants were recruited from 10 major psychiatric units located in Edmonton, Calgary, and Grand Prairie (12). Hospital patients scheduled for discharge within 7 days were recruited and invited to complete baseline surveys as part of this epidemiological investigation. Discharge decisions were determined during inpatient multidisciplinary team (MDT) meetings attended by psychiatrists, nursing staff, social workers, psychologists, and occupational therapists. The MDT reached discharge readiness decisions by consensus based on patients’ treatment responses and observed improvements in mental status. Randomization and interventions were not applied, as the study aimed to explore the prevalence and potential risk factors associated with suicidal ideation in patients nearing hospital discharge.

### 2.2 Data collection and inclusion criteria

Participant recruitment was conducted through face-to-face discussions between March 8th, 2022 to February 29th, 2024, at 10 major sites in Edmonton, Calgary, and Grand Prairie, Alberta, Canada, as part of a broader research initiative (13). Operational managers and clinical staff assisted the research team by identifying patients scheduled for discharge within 7 days from psychiatric units. Eligible participants were provided with comprehensive information about the study and invited to provide written consent. Subsequently, they completed a self-administered questionnaire on a tablet device. The survey, developed using the REDCap online platform (14), collected sociodemographic data (e.g., age, sex, ethnicity, and relationship status) and clinical information (e.g., diagnoses, anxiety and depression levels, and overall wellbeing). Clinical assessments in the study utilized validated scales for self-reported symptoms, including the Generalized Anxiety Disorder 7-item scale (GAD-7) for probable generalized anxiety disorder (GAD-7 ≥ 10) (15) Patient Health Questionnaire-9 for potential depression (PHQ-9 ≥ 10) (16), and the World Health Organization Well-Being Index (WHO-5) for poor emotional wellbeing (WHO-5 < 50) (17). Participants could choose whether researchers remained in the room during survey completion. Over the two-year recruitment period, no participants opted to complete the survey alone; many preferred researchers to stay nearby to offer immediate support if required. All eligible participants were included in the final analysis.

#### 2.2.1 Inclusion and exclusion criteria

Eligible participants were required to have a diagnosis of at least one mental illness and be scheduled for discharge from an inpatient psychiatric unit within 7 days. Additional eligibility criteria included being at least 18 years old, owning a mobile device with an active phone number, the ability to receive and read English text messages, and the capacity to provide written consent. Patients planning to leave town during the six-month follow-up period were excluded, as the main study (13) involved a peer support intervention requiring in-person meetings with peer support workers. Researchers collected participants’ phone numbers and healthcare identification numbers for tracking purposes.

### 2.3 Ethics statement

The study was approved by the Health Research Ethics Board at the University of Alberta (Ref # Pro00111459). The regional health authority also provided additional operational approval. All participants provided written informed consent.

### 2.4 Sample size calculation

In Alberta, Canada, there were 28,571 discharges from psychiatric units in 2018, with a 95% confidence interval and a ±3% margin of error, so the sample size needed for prevalence rates of likely potential suicide was 1,029 using an online script (18).

### 2.5 Outcome measures

#### 2.5.1 Patient health questionnaire-9 (PHQ-9)

The PHQ-9 is a validated self-report instrument derived from nine DSM-IV criteria for major depressive disorder (15). Participants were asked, ‘Over the past two weeks, how often have you been bothered by any of the following problems?’ Responses were scored on a four-point Likert scale, where 0 indicated ‘not at all,’ 1 represented ‘a few days,’ 2 signified ‘more than half the days,’ and 3 corresponded to ‘nearly every day.’ While the PHQ-9 is primarily employed to assess overall depressive symptomatology, individual items can be utilized to examine specific concerns, such as suicidal ideation (item 9) (19, 20).

Although the PHQ-9 is primarily used to assess depressive symptomatology, item 3 of the PHQ-9 explicitly evaluates sleep disturbances (19, 21). This item asks respondents to report the frequency of experiencing difficulty falling or staying asleep (insomnia) or sleeping too much (hypersomnia) over the past 2 weeks (21, 22). The use of PHQ-9 item 3 in this study was chosen for its established validity and efficiency in psychiatric populations, providing a practical approach to assessing sleep disturbances without introducing additional respondent burden. Previous studies have demonstrated strong correlations between PHQ-9 item 3 and comprehensive sleep measures, making it a suitable proxy for assessing sleep disturbances in this context (19, 21, 23). While the PHQ-9 sleep item encompasses both insomnia and hypersomnia, narrowing the analysis solely to insomnia would not fully capture the range of sleep disturbances observed in psychiatric populations. Therefore, we have retained the broader term “sleep disturbances” in the study, which more accurately reflects the scope of symptoms assessed.

### 2.6 Statistical analysis

Data analysis for this study was conducted using SPSS for Mac, version 25 (IBM Corp., USA) (24). Gender categories (male, female, and other) were plotted against each independent variable. Univariate analyses were performed using Chi-square tests to evaluate associations between sociodemographic and clinical factors and the categorical variable ‘trouble falling or staying asleep or sleeping too much’ (Table 1). Variables demonstrating statistical significance (*p* ≤ 0.05) or nearing significance (0.05 < *p* < 0.1) in the univariate analysis were included in the logistic regression analysis. Prior to logistic regression modeling, correlation coefficients were calculated to identify potential strong intercorrelations (Spearman’s correlation coefficient of 0.7–1.0 or −0.7 to −1.0) among predictor variables. The likelihood of experiencing sleep disturbances was quantified using odds ratios (ORs) with accompanying confidence intervals (CIs) and reported as overall percentages. Results were summarized as frequencies and percentages, with statistical significance defined at a critical level of *p* < 0.05.

**Table 1:** Chi-squared test of association between demographic and clinical characteristics and likelihood of experiencing sleep issues in the preceding 2 weeks.

Variables		Have had no 6-month ED visits *N* (%)	Have had 6-month ED visits *N* (%)	Chi square	*p*-value	Effect size
Gender	Male	123 (26.3)	345 (73.7)	19.28	<0.001^*^	0.13
	Female	100 (16.5)	505 (83.5)			
	Other	2 (6.5)	29 (93.5)			
Ethnicity	Caucasian	122 (18.0)	557 (82.0)	15.37	0.004^*^	0.12
	Indigenous	24 (23.5)	78 (76.5)			
	Black people	36 (30.0)	84 (70.0)			
	Asian	20 (16.0)	105 (84.0)			
	Mixed/other	23 (29.5)	55 (70.5)			
Relationship status	Married/partnered/common-law	47 (14.6)	275 (85.4)	13.39	0.010^*^	0.11
	Single	150 (23.0)	502 (77.0)			
	Separated or divorced	22 (25.6)	64 (74.4)			
	Widowed	3 (35.0)	9 (75.0)			
	Prefer not to say	3 (9.4)	29 (90.6)			
Primary mental health diagnosis	Depression	31 (10.5)	263 (89.5)	61.35	<0.001^*^	0.24
	Bipolar	61 (27.4)	162 (72.6)			
	Anxiety	27 (18.9)	116 (81.1)			
	Schizophrenia	61 (34.3)	117 (65.7)			
	Personality	6 (5.7)	100 (94.3)			
	Substance use	4 (26.7)	11 (73.3)			
	Other	35 (24.1)	110 (75.9)			
GAD-7	Unlikely anxiety	199 (28.6)	497 (71.4)	80.28	<0.001^*^	0.27
	Likely anxiety	24 (6.0)	377 (94.0)			
WHO-5	Good wellbeing	176 (31.1)	390 (68.9)	82.18	<0.001^*^	0.27
	Poor wellbeing	49 (9.1)	489 (90.9)			

* Significant *p* values.

### 2.7 Patient and public involvement

This study did not involve direct patient or public involvement in the study design, conduct, data analysis, or interpretation. The research was based on data collected from psychiatric inpatients at the time of discharge, with assessments conducted using validated self-report measures. However, the findings of this study have significant implications for patient care and post-discharge interventions. Future research should consider incorporating patient and public involvement to enhance study relevance and applicability, particularly in developing tailored interventions for sleep disturbances in psychiatric populations.

## 3 Results

### 3.1 Baseline data analysis

Demographic and clinical data were collected for all 1,106 participants, with gender used as a comparative variable (Table 2). The sample comprised 468 (42.3%) males, 606 (54.8%) females, with 32 (2.9%) participants identifying as other genders. Regarding age distribution, 403 (36.4%) participants were under 25 years old, 378 (34.2%) were aged 26–40, and 325 (29.4%) were over 40 years old. The majority of respondents identified as Caucasian (681, 61.6%), held a high school diploma (568, 51.4%), were unemployed (585, 52.9%), were single (652, 59.0%), and lived with family or friends (459, 41.5%). A total of 294 (26.6%) participants reported a prior diagnosis of depression. In terms of clinical conditions, 401 (36.6%) met the criteria for likely anxiety, 538 (48.7%) demonstrated poor wellbeing, and 879 (79.6%) reported experiencing sleep disturbances within the 2 weeks prior to assessment. A detailed breakdown of the respondents’ characteristics is provided in Table 2.

**Table 2:** Distribution of socio-demographic and clinical characteristics among the study participants.

Variables	Gender			
	Male (*N* = 468) *n* (%) = 42.3%	Female (*N* = 606) *n* (%) = 54.8%	Other gender (*N* = 32) *n* (%) = 2.9%	Total (*N* = 1,106)
Age				
≤25y	157 (33.5)	226 (37.3)	20 (62.5)	403 (36.4)
26–40y	168 (35.9)	199 (32.8)	11 (34.4)	378 (34.2)
>40y	143 (30.6)	181 (29.9)	1 (3.1)	325 (29.4)
Ethnicity				
Caucasian	285 (60.9)	376 (62.0)	20 (62.5)	681 (61.6)
Indigenous	33 (7.1)	65 (10.7)	4 (12.5)	102 (9.2)
Black people	54 (11.5)	64 (10.6)	2 (6.3)	120 (10.8)
Asian	54 (11.5)	68 (11.2)	3 (9.4)	125 (11.3)
Mixed/Other	42 (9.0)	33 (5.4)	3 (9.4)	78 (7.1)
Education level				
Less than high school	21 (4.5)	16 (2.6)	4 (12.5)	41 (3.7)
High school diploma	259 (55.3)	292 (48.2)	17 (53.1)	568 (51.4)
Post-secondary education	177 (37.8)	276 (45.5)	10 (31.3)	463 (41.9)
Prefer not to say	11 (2.4)	22 (3.6)	1 (3.1)	34 (3.1)
Relationship status				
Married/partnered/common-law	103 (22.0)	213 (35.1)	7 (21.9)	323 (29.2)
Single	304 (65.0)	326 (53.8)	22 (68.8)	652 (59.0)
Separated or divorced	45 (9.6)	41 (6.8)	1 (3.1)	87 (7.9)
Widowed	5 (1.1)	7 (1.2)	0 (0.0)	12 (1.1)
Prefer not to say	11 (2.4)	19 (3.1)	2 (6.3)	32 (2.9)
Employment status				
Student	30 (6.4)	56 (9.2)	3 (9.4)	89 (8.0)
Employed	130 (27.8)	186 (30.7)	12 (37.5)	328 (29.7)
Unemployed	262 (56.0)	308 (50.8)	15 (46.9)	585 (52.9)
Retired	34 (7.3)	34 (5.6)	0 (0.0)	68 (6.1)
Other	12 (2.6)	22 (3.6)	2 (6.3)	36 (3.3)
Housing status				
Own home	91 (19.4)	129 (21.3)	0 (0.0)	220 (19.9)
Rented accommodation	136 (29.1)	208 (34.3)	14 (45.2)	358 (32.4)
Live with family or friend	200 (42.7)	244 (40.3)	15 (48.4)	459 (41.5)
Couch surfing/shelter/street/other	41 (8.8)	35 (4.1)	2 (6.5)	68 (6.2)
Primary mental health diagnosis				
Depression	111 (23.7)	179 (29.5)	4 (12.9)	294 (26.6)
Bipolar disorder	86 (18.4)	132 (21.8)	5 (16.1)	223 (20.2)
Anxiety	52 (11.1)	86 (14.2)	5 (16.1)	143 (12.9)
Schizophrenia	107 (22.9)	67 (11.1)	4 (12.9)	178 (16.1)
Personality disorder	21 (4.5)	78 (12.9)	8 (25.8)	107 (9.7)
Substance use disorder	9 (1.9)	6 (1.0)	0 (0.0)	51 (1.4)
Other	82 (17.5)	58 (9.6)	5 (16.1)	145 (13.1)
GAD-7				
Unlikely anxiety	326 (69.8)	358 (59.8)	12 (38.7)	626 (63.4)
Likely anxiety	141 (30.2)	241 (40.2)	19 (61.3)	401 (36.6)
WHO-5				
Good wellbeing	274 (58.5)	284 (46.9)	8 (25.8)	566 (51.3)
Poor wellbeing	194 (41.5)	321 (53.1)	23 (74.2)	538 (48.7)
Have had difficulty falling/ staying asleep/sleeping too much in the past 2 weeks				
No	123 (26.3)	100 (16.5)	2 (6.5)	225 (20.4)
Yes	345 (73.7)	505 (83.5)	29 (93.5)	879 (79.6)

### 3.2 Univariate analysis

Table 1 presents the findings of the univariate analysis examining the association between sleep disturbances and demographic and clinical characteristics. A total of 10 variables were analyzed using Chi-squared tests, revealing that six variables were significantly associated with sleep issues. Participants identifying as genders other than male or female exhibited a higher likelihood of experiencing sleep disturbances. Similarly, individuals identifying as Asian or Caucasian were more prone to sleep disturbances compared to those from other ethnic groups, such as Black or mixed ethnicity. Regarding relationship status, individuals who chose not to disclose their status were more likely to report sleep disturbances than those who were married, single, or widowed. Moreover, participants diagnosed with personality disorders and depression were at a greater risk of experiencing sleep disturbances compared to those with other diagnoses. Finally, patients reporting moderate to severe anxiety or poor wellbeing prior to discharge demonstrated a significantly higher prevalence of sleep disturbances.

### 3.3 Logistic regression analysis results

We entered the six variables that were identified by the univariate analysis as statistically significant into the logistic regression model to predict the likely sleep issues. The overall model was statistically significant, *X*^2^(18) = 200.50, *p* < 0.001, indicating that it effectively differentiated between participants who did and did not exhibit sleep disturbances prior to hospital discharge. The model explained 16.7% (Cox and Snell R^2^) and 26.3% (Nagelkerke R^2^) of the variance and accurately classified 80.1% of cases. As detailed in Table 3, three variables including primary mental health diagnosis, likely anxiety, and baseline wellbeing were found to significantly predict sleep disturbances before discharge. While the relationship status variable did not contribute significantly to the model, it was found that the participants who were single individuals (OR = 0.66; 95% CI: 0.44–0.98) and those who were separated or divorced (OR = 0.43; 95% CI: 0.22–0.83) were less likely to endorse sleep disturbances compared to the participants who were identified as married, partnered, or in a common-law relationship. This means that the participants who were identified as married, partnered, or in a common-law relationship were approximately twice as likely to report sleep disturbances compared to the two other groups. Furthermore, compared to the participants who were diagnoses with depression, individuals who were diagnosed with bipolar disorder (OR = 0.40; 95% CI: 0.24–0.66), schizophrenia (OR = 0.32; 95% CI: 0.19–0.54), and other diagnoses (OR = 0.52; 95% CI: 0.29–0.92) were around two to three times less likely to develop sleep disturbances. Participants meeting the criteria for generalized anxiety disorder (GAD) were over four times more likely to report sleep disturbances compared to those with mild anxiety (OR = 4.50; 95% CI: 2.81–7.21). Additionally, individuals with poor wellbeing at discharge were nearly three times more likely to experience sleep disturbances compared to those with good wellbeing (OR = 2.67; 95% CI: 1.84–3.89), after accounting for other factors.

**Table 3:** Logistic regression predicting the likelihood of residents presenting sleep issues.

Variables		*B*	S.E.	Wald	df	*p*-Value	Odd’s ratio	95% C.I. for EXP(B)	
								Lower	Upper
Gender	Male			2.464	2	0.292			
	Female	0.219	0.174	1.592	1	0.207	1.245	0.886	1.751
	Other gender	0.821	0.774	1.126	1	0.289	2.273	0.499	10.359
Ethnicity	Caucasian			7.593	4	0.108			
	Indigenous	−0.460	0.292	2.483	1	0.115	0.631	0.356	1.119
	Black	−0.406	0.254	2.565	1	0.109	0.666	0.405	1.095
	Asian	0.292	0.285	1.045	1	0.307	1.339	0.765	2.343
	Mixed/other	−0.396	0.305	1.686	1	0.194	0.673	0.370	1.223
Relationship status	Married/partnered/common-law			8.703	4	0.069			
	Single	−0.422	0.204	4.286	1	0.038*	0.656	0.440	0.978
	Separated or Divorced	−0.845	0.333	6.452	1	0.011*	0.430	0.224	0.825
	Widowed	−0.367	0.763	0.232	1	0.630	0.693	0.155	3.088
	Prefer not to say	0.344	0.669	0.264	1	0.608	0.1.410	0.380	5.237
Primary mental health diagnosis	Depression			29.235	6	<0.001*			
	Bipolar	−0.923	0.260	12.637	1	<0.001*	0.397	0.239	0.661
	Anxiety	−0.554	0.309	3.206	1	0.073	0.575	0.313	1.054
	Schizophrenia	−1.153	0.271	18.181	1	<0.001*	0.316	0.186	0.536
	Personality	0.583	0.480	1.479	1	0.224	1.792	0.700	4.590
	Substance use	−0.800	0.663	1.457	1	0.227	0.449	0.122	1.647
	Other	−0.661	0.293	5.099	1	0.024*	0.516	0.291	0.916
GAD-7 at baseline	Likely anxiety	1.504	0.240	39.174	1	<0.001*	4.501	2.810	7.209
WHO-5 at baseline	Poor wellbeing	0.983	0.192	26.295	1	<0.001*	2.673	1.836	3.893
Constant		1.527	0.286	28.451	1	<0.001	4.605		

C.I., Confidence interval; S.E., Standard error; Df, Degree of freedom. *Significant predictor.

## 4 Discussion

This study examined the prevalence and potential predictors of sleep disturbances among patients shortly after discharge from psychiatric units throughout Alberta, Canada. The study found that 879 (79.6%) of participants reported experiencing sleep disturbances within 2 weeks of leaving psychiatric hospitals. This finding aligns with previous research indicating that between 50 and 90% of patients with mental health disorders suffer from chronic sleep disturbances (10, 25–27). Research on sleep issues among psychiatric inpatients at discharge is limited. However, one study found that patients with poor sleep quality were more likely to be readmitted than those with good sleep quality (28). Another study, though not focused on psychiatric units, found that among 55 critically ill patients, 62% experienced poor sleep quality before and 3 months after hospital discharge (29).

Although relationship status did not significantly contribute to the model, this study observed that single individuals tend to have better sleep quality than those who are married or partnered. This aligns with research suggesting marital status influences sleep and broader health outcomes (30–32). Some studies suggest that married individuals experience more family responsibilities, leading to sleep sacrifices (30–34), or sleep disturbances from a partner’s condition, such as sleep apnea (35). However, other research suggests marriage has a protective effect, improving overall health and potentially sleep quality (36–38).

Depression was a strong predictor of sleep disturbances, consistent with extensive prior research (39–45). Studies indicate that nearly three-quarters of depressed individuals struggle with sleep initiation or maintenance (40, 42, 43). One UK-based study found that 83% of those with depression reported at least one insomnia symptom, compared to only 36% of those without depression (45). Further research from the United States highlights that approximately 40% of individuals with insomnia meet the criteria for clinical depression, while up to 80% of those diagnosed with depression report significant sleep difficulties (39). The link between depression and sleep disturbances may be explained by neurotransmitter dysregulation (46), particularly in serotonin, norepinephrine, and dopamine (46, 47), and disruptions in the hypothalamic–pituitary–adrenal (HPA) axis, which is critical for sleep regulation (48–50).

Symptoms of anxiety and poor wellbeing also significantly predicted sleep disturbances. Mild anxiety can serve as an internal alarm, helping individuals respond to potential threats. However, in anxiety disorders, the individual experiences false alarms that are often intense, frequent, or persistent (51). These false alarms can result in dysfunctional arousal, frequently contributing to ongoing sleep–wake difficulties (52). Surveys indicate that 24–36% of individuals with sleep difficulties also have an anxiety disorder, and individuals with panic disorder are three times more likely to report sleep issues (51). Research suggests a bidirectional relationship: sleep disturbances can exacerbate anxiety, while pre-existing anxiety disorders can contribute to sleep difficulties (29, 53). Neuroimaging studies show that individuals with anxiety exhibit heightened activity in the amygdala and insula, regions involved in emotional regulation, and may contribute to increased emotional vulnerability to sleep deprivation (54). While much research focuses on how sleep affects wellbeing, fewer studies explore the reverse relationship. Positive wellbeing is linked to health-protective factors such as lower cortisol levels, reduced cardiovascular stress responses, and decreased mortality in older populations (55, 56). Chronic stress, poor emotional support, and social isolation are associated with diminished wellbeing and increased sleep disturbances (57–60).

Based on the study findings, several targeted interventions could be developed to address the high prevalence of sleep disturbances among psychiatric patients at discharge. Given that depression, anxiety and poor wellbeing, were both identified as significant predictors of sleep issues, interventions should focus on enhancing coping strategies for stress, promoting relaxation techniques, and improving overall emotional wellbeing. For instance, cognitive-behavioral therapy for insomnia (CBT-I) is a well-established intervention that could be integrated into discharge planning to help patients learn how to manage thoughts that interfere with sleep (61). CBT-I has been demonstrated to reduce insomnia symptoms effectively, even in individuals with comorbid mental health conditions (61, 62). Mindfulness-based interventions (MBIs), such as mindfulness-based stress reduction (MBSR), could also be beneficial in promoting relaxation and reducing anxiety (63, 64). MBSR has shown effectiveness in improving sleep quality and reducing symptoms of anxiety and depression, which makes it a suitable option for patients in transitional care phases (64). Additionally, psychoeducation sessions focusing on sleep hygiene and stress management techniques could help patients build habits conducive to better sleep post-discharge (65). Support from healthcare professionals during discharge can also be enhanced by regular follow-up sessions, either in person or virtually, where sleep patterns can be monitored, and adaptive strategies reinforced (66). Peer support groups and some innovative supportive interventions such as Text4Support could also play a role in helping patients share their experiences and gain professional support, which could reduce anxiety and depression and improve emotional wellbeing (67, 68). Finally, involving family members in the intervention process, by educating them on the importance of supportive environments for sleep health, could further aid in mitigating sleep disturbances for patients post-discharge (69).

## 5 Limitations

This study has several limitations that should be acknowledged. Firstly, it relied exclusively on self-reported measures, including the GAD-7, PHQ-9, and WHO-5 Well-Being Index, which may introduce response biases and compromise the objectivity of the findings. The assessment of sleep disturbances was based on a single item from the PHQ-9, rather than a more comprehensive and validated sleep-specific scale such as the Pittsburgh Sleep Quality Index (PSQI). While the PHQ-9 sleep item has demonstrated validity in screening for sleep issues in psychiatric populations, it does not capture the full complexity of sleep quality. Future studies should consider incorporating objective measurement tools, such as actigraphy or polysomnography, to provide a more accurate and nuanced understanding of sleep disturbances. Additionally, the generalizability of the findings is limited to the specific healthcare and demographic context of Alberta, Canada. The study’s participants were recruited from psychiatric units in this region, which may not reflect the experiences of individuals in other geographic areas or healthcare systems. To enhance the applicability of future research, it is recommended to include diverse populations across different regions and healthcare settings. Moreover, the study did not collect detailed information about hospital services or patient care, such as treatment types, duration, or mental state upon admission, which might have provided additional insights into factors influencing post-discharge sleep disturbances. Medical factors such as substance use and physical health status, which could act as confounding variables, were also not comprehensively assessed. Lastly, the cross-sectional design of the study precludes causal inferences, making it difficult to determine whether the identified predictors directly contribute to sleep disturbances. Longitudinal studies are needed to explore the temporal relationships and causal pathways between these variables. Despite these limitations, the study contributes novel information from a large cohort which may help our understanding of sleep disturbances in psychiatric patients around the time of discharge, highlighting key predictors and underscoring the importance of addressing these issues during the transition to community care.

## 6 Conclusion

In this study, the prevalence and potential risk factors associated with sleep issues were assessed among Albertan patients who were about to be discharged from psychiatric units. In total, 879 (79.6%) of participants reported experiencing sleep disturbances within 2 weeks before filling out the survey. It was found that the married/in relationship status, depression diagnoses, likely anxiety, and poor wellbeing at baseline were significant predictors of participants’ potential sleep issues upon discharge from the hospital. These findings emphasize the critical need to address sleep disturbances as an integral component of discharge planning and post-discharge care for psychiatric patients. The identified predictors offer valuable insights into the multifaceted factors contributing to sleep issues within this population, highlighting the importance of a comprehensive approach to care. Implementing targeted interventions, including individualized mental health support, psychoeducation, and evidence-based sleep-focused therapies, holds the potential to significantly improve recovery trajectories and enhance overall quality of life. Future research should prioritize longitudinal studies to evaluate the sustained impact of these interventions on mitigating sleep disturbances and promoting mental health stability following hospital discharge.

## Data Availability

The original contributions presented in the study are included in the article/Supplementary material, further inquiries can be directed to the corresponding author.
