# Supplementary material for: The prevalence and risk factors of sleep disturbances among mental health patients following hospital discharge
Source: Front Public Health. 2025 Jul 30;13:1595303. doi: 10.3389/fpubh.2025.1595303 (PMC12343569; doi:10.3389/fpubh.2025.1595303)
Supplement: Supplementary file 1 [file Table_1.docx]

Appendix I:

Text4Support plus peer support project team (T4SPPSP-Team)

| **Last Name** | **First Name** | **Primary Organization** | **Organizational Position** |
| --- | --- | --- | --- |
| Agyapong | Vincent | Department of Psychiatry, Dalhousie University | Professor and Head |
| Bales | Kerry | Addiction and Mental Health Strategic  Clinical Network | Senior Program Officer |
| Taylor | Valerie | Department of Psychiatry, University of  Calgary | Head |
| Greenshaw | Andrew | Department of Psychiatry, Faculty of  Medicine and Dentistry, University of  Alberta | Associate Chair |
| Ohinmaa | Arto | School of Public Health, University of  Alberta and Institute of Health Economics | Professor |
| Hilario | Carla | Faculty of Nursing, University of Alberta | Assistant Professor |
| Greiner | Russ | Faculty of Science, University of Alberta | Professor |
| Silverstone | Peter | Department of Psychiatry, University of  Alberta & Edmonton Zone Community  Mental Health | Professor |
| McCabe | Christophe | Institute for Health Economics | Executive Director/ CEO |
| Bremault Phillips | Suzette | Heroes in Mind, Advocacy and Research  Consortium (HiMARC)/ University of  Alberta | Director HiMARC and Associate  Professor |
| Cao | Bo | Department of Psychiatry, Faculty of  Medicine and Dentistry, University of  Alberta | Assistant Professor |
| MacMaster | Frank | Addiction and Mental Health Strategic  Clinical Network | Scientific Director |
| Bales | Kerry | Addiction and Mental Health Strategic Clinical Network | Senior Program  Officer |
| Snaterse | Mark | Addiction and Mental Health, Alberta Health Services,  Edmonton Zone | Executive  Director |
| Chafe | Janet | Addiction and Mental Health, Alberta Health Services,  Calgary Zone | Executive  Director |
| Greening | Stacy | QEII Regional Hospital and North Zone, Addictions and  Mental Health Alberta Health Services | Senior Operating  Officer |
| Given | Susan | Addiction and Mental Health, Alberta Health Services,  North Zone | Executive  Director |
| McLean | Carla | Addiction and Mental Health, Alberta Health Services,  North East Zone | Director |
| Rittenbach | Katherine | Addiction and Mental Health Strategic Clinical Network | Assistant  Scientific  Director |
| Li | Xin-Min | Department of Psychiatry, Faculty of Medicine and  Dentistry, University of Alberta | Chair |
| Rathwell | Rebecca | Edmonton Zone Peer Support Practice Council | Co-chair |
| Knox | Michelle | Housing and Recovery Supports Young Adult and  Cross Level Services Addiction and Mental Health,  Edmonton Zone | Program manager |
| Chue | Pierre | Department of Psychiatry, Faculty of Medicine and  Dentistry, University of Alberta | Clinical Professor |
| Abda-Aji | Adam | Department of Psychiatry, Faculty of Medicine and  Dentistry, University of Alberta | Associate Clinical  Professor |
| Surood | Shireen | Information, Evaluation & Professional Practice  Addiction & Mental Health, Alberta Health Services | Manager |
| Nwachukwu | Izu | Department of Psychiatry, University of Calgary | Associate Clinical  Professor |
| Vuong | Wesley | Decision Support Services - Business Intelligence  Information, Evaluation & Professional Practice  Addiction & Mental Health - Edmonton Zone | Research & Evaluation  Coordinator |
| Shalaby | Reham | Department of Psychiatry, Faculty of Medicine and  Dentistry, University of Alberta | PhD Candidate |
| Berhe | Tzeggai | QEII Regional Hospital and North Zone, Addictions and  Mental Health Alberta Health Services | Zone Clinical Facility  Chief |
| Ambrosano | Lorella | Northern Lights Regional Health Centre | Zone Clinical Facility  Chief for Addiction and  Mental Health |
| Grauwiler | David | Canadian Mental Health Association, Alberta | Executive Director |
| Jordan | Sara | Canadian Mental Health Association, Calgary Zone | Executive Director |
| Lindy | Fors | Patients' Advisory Council, Addiction and Mental Health,  Alberta Health Services | Patient Advisor |
| Brown | Ed | Patients' Advisory Council, Addiction and Mental Health,  Alberta Health Services | Patient Advisor |
| Savard | Tyla | Patients' Advisory Council, Addiction and Mental Health,  Alberta Health Services | Patient Advisor |
| Spurvey | Pamela | Addiction and Mental Health, Alberta Health Services,  Edmonton Zone | Peer support worker |
| Grauwiler | David | Canadian Mental Health Association, Alberta  Division | Executive Director |
| Grunau | Mara | Centre for Suicide Prevention | Executive Director |
| Frank | Kelton | Potential Place | Executive Director |
| Sheila | Stauffer | Cornerstone Counselling | Executive Director |
